# Single versus multiple imputation for genotypic data

**DOI:** 10.1186/1753-6561-3-s7-s7

**Published:** 2009-12-15

**Authors:** Brooke L Fridley, Shannon K McDonnell, Kari G Rabe, Rui Tang, Joanna M Biernacka, Jason P Sinnwell, David N Rider, Ellen L Goode

**Affiliations:** 1 Department of Health Sciences Research, Mayo Clinic, 200 First Street Southwest, Rochester, MN 55905, USA

## Abstract

Due to the growing need to combine data across multiple studies and to impute untyped markers based on a reference sample, several analytical tools for imputation and analysis of missing genotypes have been developed. Current imputation methods rely on single imputation, which ignores the variation in estimation due to imputation. An alternative to single imputation is multiple imputation. In this paper, we assess the variation in imputation by completing both single and multiple imputations of genotypic data using MACH, a commonly used hidden Markov model imputation method. Using data from the North American Rheumatoid Arthritis Consortium genome-wide study, the use of single and multiple imputation was assessed in four regions of chromosome 1 with varying levels of linkage disequilibrium and association signals. Two scenarios for missing genotypic data were assessed: imputation of untyped markers and combination of genotypic data from two studies. This limited study involving four regions indicates that, contrary to expectations, multiple imputations may not be necessary.

## Background

Due to the growing need to combine data across multiple studies, several analytical tools for imputation and analysis of missing genotypes have been developed and assessed [1-4]. These methods are particularly useful in the context of failed genotyping and combining data across multiple platforms, and recently have been extended to untyped markers using a reference sample [2-4]. Current imputation methods typically rely on single imputation (SI); however, SI ignores the variation in estimation due to the imputation. Therefore, one is unable to determine the variation in association results due to the imputation technique.

An alternative to SI is multiple imputation (MI) in which multiple imputed or "augmented" datasets are created and then analyzed using standard statistical methods and models [5,6]. In this paper, we compare the use of SI and MI using the software MACH [4] to impute genotype "dosage" between 0 and 2. In a companion Genetic Analysis Workshop (GAW) 16 analysis, we assessed four commonly used imputation packages (MACH [4], fastPHASE [1], IMPUTE [2], PLINK [7]) and concluded that using MACH or IMPUTE led to the lowest imputation error rates [8], consistent with other reports that MACH and IMPUTE yield similar imputation accuracy [9,10]. We chose to use MACH rather than IMPUTE for this comparison of SI versus MI because MACH required less memory to run, and we considered it to be more "user-friendly". The comparison of SI and MI was completed using the North American Rheumatoid Arthritis Consortium (NARAC) data [11]. We examine the variation in imputation and implication on association results.

## Methods

### Data

**Figure 1:**
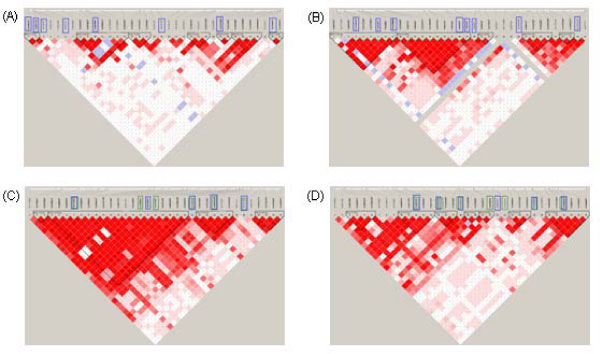
**Four genomic regions**. (A) Null region 1 - low LD; (B) Null region 2 - high LD; (C) Associated region - *PTPN22 *(D) Associated region - *PADI4*. SNPs in boxes indicate those removed for imputation of completely untyped markers. For associated regions (A and B), boxes indicate SNPs which were imputed: blue boxes denote the set containing the imputed risk SNP (first SNP set) and green boxes denote the set including flanking markers to the risk SNP (second SNP set).

The NARAC data consists of 868 cases of rheumatoid arthritis (RA) and 1194 controls genotyped on the 550 k Illumina single-nucleotide polymorphism (SNP) chip [11,12]. To mimic a variety of genetic models, we assessed four regions on chromosome 1 (two with positive associations, two with null associations). Associated regions included *PTPN22*, which has been reported to harbor the risk SNP rs2476601 [13,14] and *PADI4*, which also has a reported risk allele for RA [15], in which *PADI4 *displays lower linkage disequilibrium (LD) than *PTPN22*. Two null regions on chromosome 1 were also selected: one with high LD and one with low LD (Figure 1). Before analysis, SNPs deviating from Hardy-Weinberg equilibrium (HWE) (*p *< 0.001) or with call rates <95% were removed.

### Single and multiple imputation

Analyses under two scenarios were completed; for both scenarios, we have "true" genotypes. Scenario I mimicked the situation in which completely untyped markers were imputed. In this scenario, a set of SNPs genotyped in the NARAC cohort were selected to be removed based on various criteria (e.g., minor allele frequency (MAF), significance, LD) and were then imputed in the entire cohort. For both 'associated' regions (Figure 1, C and 1D), two sets of SNPs were imputed, resulting in a total of six datasets for analysis (two for each associated region, one for each null region). The risk SNP was defined as the SNP with the strongest evidence of association (rs2476601 in *PTPN22*, rs6683201 in *PADI4*). In the first set, the risk SNP was imputed; in the second set, the two markers flanking the risk SNP were imputed.

Scenario II mimicked the situation in which two studies genotyped different set of SNPs; 1/3 of the SNPs were genotyped only in Study I, 1/3 of the SNPs were genotyped only in Study II, and the remaining 1/3 of the SNPs were genotyped in both Study I and Study II. We created the two studies by randomly splitting the NARAC data, ensuring equal numbers of cases and controls in each study. Likewise, the SNPs were randomly chosen to be genotyped in Study I, Study II, or both studies.

Each of the four regions for both scenarios were analyzed five times using MACH version 1.0.16 [4] with phased HapMap haplotypes for the 60 CEU founder participants as the reference haplotypes [16]. For each region, 150 iterations were used to insure convergence, where minor allele "dosage" (expected mean genotype) was imputed. Syntax used for running MACH was the following: mach1 -d region.dat -p region.ped -h region.haplos -s region.snps --rounds 150 --greedy --geno --dosage --quality --mask 0.02 --seed 487 > mach.out.

Associations between SNP genotypes and RA risk were then assessed using logistic regression to estimate odds ratios (ORs), 95% confidence intervals (CIs), and *p*-values. Tests for association assumed an ordinal (log-additive) genotypic effect on RA risk. Inference for parameters from multiple imputations was completed as follows: let *θ *represent the parameter of interest, and *K *represent the number of imputed datasets (e.g., *K *= 5). The overall point estimate of *θ *is the mean of the *K *point estimates based on the imputed datasets. The estimated variance of  is defined as , where *W *and *B *represent the within and between imputation variation. Inference for *θ *is then based on the *t*-distribution with *df *= (*k *- 1)(1 + (1/(*K *+ 1))(*W*/*B*))^2 ^[5].

## Results

**Figure 2:**
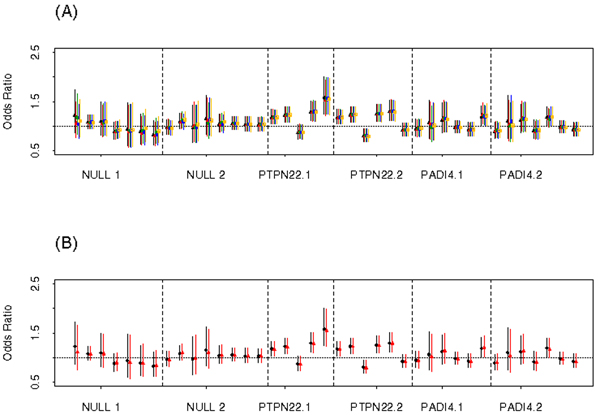
**(A) Odds ratios with 95% CI for each of the 5 imputation runs; (B) Odds ratios with 95% CI based on SI and MI from MACH Scenario I**.

Comparison of the standard error for the SNP coefficient from logistic regression between SI (first run or run 1) and MI revealed only a small differences in the standard error and ORs between SI and MI, as illustrated with the 95% CIs for the ORs from SI and MI for Scenario I (Figure 2B). The median difference in standard error between SI and MI was -0.0004 and -0.0001 for Scenario I and II, respectively. The median difference in ORs was 0.0005 with and IQR (interquartile range) of 0.008 for Scenario I, while the median difference in ORs was 0.0002 with an IQR of 0.006 for Scenario II.

In terms of impact on testing for association using SI and MI, results were very similar between SI and MI. For Scenario I, the median difference in -log_10_(*p*-values) was 0.005 with IQR of 0.077, while for Scenario II, the median difference was 0.002 with and IQR of 0.044. Scenario I had slightly greater variation in *p*-values between SI and MI as compared to Scenario II. Next, we evaluated the variation in imputed genotypes from two imputation runs (run 1 and run 2), summarized by SNP and by subject, for Scenario I and II. The median difference in imputed genotypes, summarized by SNP, was 0.0002 (IQR = 0.002) and 0.0003 (IQR = 0.001) for Scenario I and II, respectively. The median difference (IQR) between imputed genotypes, when summarized by subject, for Scenario I and II was 0.0005 (IQR = 0.011) and 0.0003 (IQR = 0.007).

**Figure 3:**
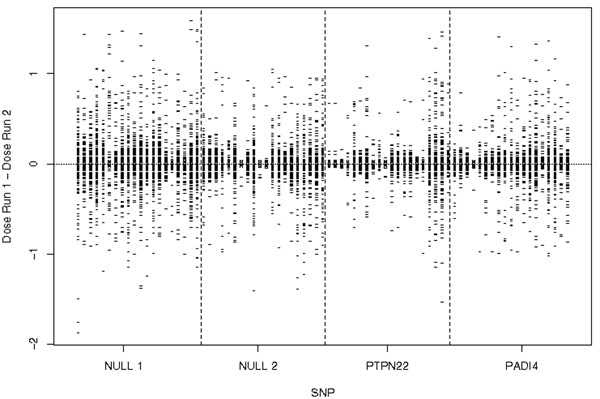
**Box plots of difference in individuals' dosage (observed or imputed) for each of the SNPs with missing data (Scenario II) from MACH**.

LD had a greater effect on variation in imputation in regions of low LD, as expected. For example, the variation in imputation of genotypes from MACH was larger in the null 1 region (low LD) as compared to the null 2 region (high LD) (Figure 3). We also observed that variation in ORs from association analyses completed on multiple imputed datasets was higher in regions of low LD (Figure 2A).

## Discussion and conclusion

We have demonstrated the use of SI and MI for the imputation of missing genotypes or untyped markers using a reference panel. In doing so, we utilized MACH [4], a common method that relies on LD and haplotype estimation via a hidden Markov model. A companion GAW16 paper assessed four commonly used imputation packages and concluded that using MACH or IMPUTE led to the lower imputation error rates than using fastPHASE or PLINK [8]. Care should be taken to select the most appropriate imputation method as well as to determine whether to use SI or MI.

Another consideration of whether one should employ MI is computation time. For the analyses presented, MACH was run on a Beowolf-style Linux cluster with compute nodes running CentOS 4.3 Linux x86-64 allowing 8-16 GB memory per job. Scenario I run-times (single imputation) ranged from 13-18 minutes. However, when MACH used the raw genotype data for the reference samples instead of the phased haplotypes, the run-time increased to more than 30 days [mach1 -d regionPool.dat -p regionPool.ped --rounds 150 --compact --geno --dosage --quality --mask 0.02 --seed 1776 > mach.out]. In contrast, the run-times for Scenario II, based on phased haplotypes, was around 10 minutes with little variation in run times between the four regions.

Ignoring variation due to imputation results in under-estimation of the variance in the parameter estimate, and hence an inflated type I error. For imputation of untyped markers (Scenario I), we observed larger variation in results as compared to imputation of missing genotypes (Scenario II). For Scenario II, we observed small differences in association results based on SI or MI, especially in regions of higher LD. In genome-wide association studies in which SI is often implemented for over two million markers, one appropriate approach is to use SI as the initial analysis and employ MI for any regions of interest detected with SI to assess variation due to imputation. On the basis of this study involving four regions, single imputation is reasonable, especially in regions of high LD where imputed genotype "dosage" is used in the analysis.

## List of abbreviations used

GAW: Genetic Analysis Workshop; HWE: Hardy-Weinberg equilibrium; IQR: Interquartile range; LD: Linkage disequilibrium; MAF: Minor allele frequency; MI: Multiple imputation; NARAC: North American Rheumatoid Arthritis Consortium; ORs: Odds ratios; RA: Rheumatoid arthritis; SI: Single imputation; SNP: Single-nucleotide polymorphism.

## Competing interests

The authors declare that they have no competing interests.

## Authors' contributions

BLF conceived of the study and coordinated it. BLF, JMB, ELG, SKM, KGR, RT, JPS, and DNR participated in design of study. SKM, KGR, RT, and JPS ran statistical analysis. BLF, ELG, and JMB drafted manuscript. All authors read and approved the final manuscript.
